# Representative mammalian cell culture test materials for assessment of primary recovery technologies: A rapid method with industrial applicability

**DOI:** 10.1002/biot.201400294

**Published:** 2014-11-06

**Authors:** Daria Popova, Adam Stonier, David Pain, Nigel J Titchener-Hooker, Suzanne S Farid

**Affiliations:** 1 Department of Biochemical Engineering, University College London London, UK; 2 Lonza Biologics plc Slough, Berkshire, UK

**Keywords:** Bioseparation, Mammalian cell culture, High cell density, Primary recovery

## Abstract

Mammalian cell culture material is often difficult to produce accurately and reproducibly for downstream studies. This article presents a methodology for the creation of a set of cell culture test materials where key variables including cell density, cell viability, product, and the host cell protein (HCP) load can be manipulated individually. The methodology was developed using a glutamine synthetase Chinese hamster ovary cell line cultured at 5-L and 70-L scales. Cell concentration post-cell growth was manipulated using tangential flow filtration to generate a range of target cell densities of up to 100 × 10^6^ cells/mL. A method to prepare an apoptotic cell stock to achieve target viabilities of 40–90% is also described. In addition, a range of IgG_1_ and HCP concentrations was achieved. The results illustrate that the proposed methodology is able to mimic different cell culture profiles by decoupling the control of the key variables. The cell culture test materials were shown to be representative of typical cell culture feed material in terms of particle size distribution and HCP population. This provides a rapid method to create the required feeds for assessing the feasibility of primary recovery technologies designed to cope with higher cell density cultures.

## 1 Introduction

Mammalian cell culture titers and cell densities have been increasing over the years driven by product demand, cost efficiency targets, and production facility constraints. Cell culture titers have been reported to have reached >13 g/L in fed-batch cultures [1], with cell densities beyond 20 × 10^6^ cells/mL [2]. These rises have contributed to an increase in pressure on primary recovery and purification operations, which have to adapt to changes in the feed stream. Although research into alternative downstream technology options has been carried out over the years [3], there has been little focus on how to generate representative feed materials for experimentation.

The key variables, which define the properties of feeds to primary recovery operations tend to be specific to the operation in question. However, most published studies have historically included factors such as solids load [4], cell density and cell viability [5], and more specifically particle size distribution [6]. The impact of primary recovery and purification operations on product recovery as well as DNA and host cell protein (HCP) impurity removal have been discussed [2], most often in the context of cell line and cell culture conditions [7].

Research focused on increasing the understanding of primary recovery technologies such as centrifugation [8, 9], depth filtration [10, 11], and tangential flow filtration (TFF) options [12], have typically used cell culture material harvested directly from bioreactors. In these cases harvest conditions have been stated, such as day of harvest, cell density at harvest or viability at harvest, but little evidence of control over these feedstream conditions are shown. Some studies have focused on just one of the variables, for example cell viability, and had material harvested at different days from the cell culture bioreactor to obtain feed material with varying levels of viability [6]. However, adopting such a methodology can make it difficult to contextualize the results when relating to other cell lines or cell culture processes. For example at identical cell culture days different cell lines or processes can yield a range of viabilities. In addition unique cell death pathways can result in differing particle size distributions being passed onto the primary recovery operations [13].

Spiking of impurities as well as product has also been used in the past to mimic different levels of impurity loads [14, 15] or to provide a measurable concentration, which can be quantified prior to and post-technology or condition implementation. However, the stability of the spiked material in solution must be verified to ensure the spiked material can be accurately quantified.

Comparing the cell culture material produced using different cell lines for primary recovery studies and purification has also been previously shown [2, 5]. However, this method can have limitations when deriving correlations or empirical models, as potential biological expression differences can create a large set of unknowns, especially where HCP profile differences are significant.

The level of control required to achieve specific properties in cell culture feeds to downstream operations can cause considerable strain on the cell culture material generation even prior to the material reaching primary recovery stages. Other variables can also contribute to the number of unknowns which in turn could potentially affect the downstream experimentation including cell culture scale and scale up methodology, feeding strategies, and metabolite profiles during processing.

This article describes a novel methodology for the consistent creation of materials with a defined set of process-relevant characteristics and is aimed at producing material for primary recovery studies. The approach is to control tightly key cell culture variables and to ensure they are maintained independently so as to decouple effects from one another. Such an approach is difficult to achieve when using cell culture material sourced directly from a cell culture operation. The methodology aims to increase the level of information, which can then be derived from carrying out primary recovery and purification studies and to provide a new avenue for deriving empirical performance correlations for novel processing technologies, in particular those designed to handle high cell density feeds.

## 2 Materials and methods

### 2.1 Cell culture 5-L scale

Cell culture material was generated using an IgG_4_ producing cell line (CY01) kindly provided by Lonza Biologics (GB-Slough, Berkshire, UK) in a 5-L (3.5-L working volume) bioreactor with a stirred tank reactor (STR) with an in-built control system (B. Braun BIOSTAT B-DCU control unit, Sartorius, Epsom, UK). Set points were maintained at 30% air dissolved oxygen tension (DO), 7.10 pH and a temperature of 37°C. A constant gas flowrate of 100 cm^3^/min was maintained using a horseshoe sparger. Agitation was set at 260 RPM using a single 45° pitch, three-blade impeller. All cell cultures were carried out in fed-batch mode using chemically defined medium (CDCHO, Invitrogen, Paisley, UK). The glucose concentration was measured daily with a NOVA Bioanalyser (Nova Biomedical, Deeside, UK) and maintained at a concentration of 2 g/L using a bolus feed of ten-fold concentrated dry powder CD CHO media, adjusted to a concentration of 150 g/L glucose (Sigma, Poole, Dorset, UK).

### 2.2 Cell culture 70-L scale

The CY01 cell line was also used to produce material at the 70-L scale using a single-use bioreactor (SUB) (BIOSTAT CultiBag STR, Sartorius, UK). Set points were maintained at 30% dissolved oxygen and a pH of 7.1 at 37°C. Agitation rate was set at 150 RPM. Cell culture media and feeding strategy were matched to the 5-L STR conditions.

### 2.3 Shear study comparison

Cell culture material was harvested at the 5-L scale once the viability had reached approximately 75%. The material was loaded into a rotating shear device [4, 6, 9, 16]. High and low shear (equivalent to maximum energy dissipation rates of 0.37 × 10^6^ and 0.019 × 10^6^ W/kg, respectively) were applied to the material, with each condition examined in triplicate. Particle size distributions obtained pre- and post-exposure to high and low shear of each material batch were normalized and compared.

### 2.4 Cell culture harvest

All 5-L STR harvests were performed on days 13–14 of culture, once the viability had declined to ∼70%. The 70-L STR harvests were performed daily between day 7 and day 14 of culture, where 3L of cell culture material was removed per daily harvest.

### 2.5 Induced cell apoptosis

On day 13 of the cell cultures, approximately 300 mL of cell culture material was removed from the 5-L STR, aliquoted into 50-mL falcon tubes and washed with a sterile phosphate buffered saline solution (PBS, Sigma–Aldrich, Gillingham, Dorset, UK). The sedimented cells were then incubated on ice for 2 h, then maintained at 37°C for up to 24 h prior to use. Samples were monitored in the first 6 h post-apoptosis induction by staining using the Annexin V-FITC/7ADD kit PN IM3546 (Beckman Coulter, High Wycombe, UK) and processed using a Coulter Epics XL-MCL Flow Cytometer (Beckman Coulter, High Wycombe, UK). Apoptosis data were collected and analyzed using EXPO 32 ADC XL Color software (Beckman Coulter, High Wycombe, UK). Samples were analyzed using 488 nm excitation and detected using 525 and 675 nm band-pass filters for Annexin V and 7-ADD (7-amino-actynomycin D), respectively, and collected for 300 s (10000 events). On the day of harvest the required volume of the dead cell stock was calculated based on cell density and viability measurements of both the harvest material and the dead cell stock, obtained using a ViCell™ (Invitrogen, Paisley, UK).

### 2.6 Cell concentration

Cell culture material was concentrated post-harvest, using an Asahi Kasei Bio-Optimal MF SL™ module, area 0.005 m^2^ (Asahi Kasei Medical Co., Tokyo, Japan). The module was run in TFF mode using the AKTA crossflow system (GE Healthcare, Little Chalfort, UK) as well as a manually controlled peristaltic pump for permeate flow control. The feed was circulated at 240 mL/min and permeate was collected at a constant flowrate of 3.5 mL/min, until the target concentration factor was achieved. The starting and final concentration volumes (*V*_S_ and *V*_C_, respectively, in Eqs. 1 and 2) were calculated based on the starting (*D*_S_) and desired (*D*_R_) cell densities, as well as the spike volumes of the product (IgG_SP_ and impurity spikes (HCP_SP_, DNA_SP_) and the dead cell stock spike (DC_SP_) required.


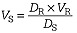
(1)


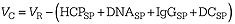
(2)

### 2.7 Impurity and product concentration

Fully purified IgG_1_ and flowthrough fraction post-protein A chromatography was kindly provided by Lonza Biologics. The flowthrough fraction was used to produce the HCP spike stock solution while the fully purified IgG_1_ was used to create the product stock solution. Both the HCP and IgG_1_ fractions were concentrated to approximately 50 g/L using a Vivaspin® 20 (Sartorius, UK) with 5000 and 50000 MWCO filters, respectively. Post-treatment concentrations of the HCP stock were confirmed using the BCA assay (Fisher Scientific, Loughborough, UK). The IgG_1_ was quantified by measuring absorbance at 280 nm.

### 2.8 Analytical techniques

#### 2.8.1 Cell density and viability measurements

Cell culture density and viability were determined using the ViCellTM (Beckman Coulter, High Wycombe, UK). Consistency in cell density measurements is dependent on the dilution required prior to the measurement. Cell density replicates were typically within ±4 × 10^6^ cells/mL, however at higher cell densities standard deviations were up to 10 × 10^6^ cells/mL due to higher dilution factor requirements.

#### 2.8.2 Particle size distribution

Particle size distribution measurements were obtained using the CASY™ (Innovatis, Bielefeld, Germany) analyzer. A 150 μm sized orifice was used, providing a measured particle size range of 0.2–40 μm. Average measurements of the mean particle size (solids volume basis) were used, reported from five analyzer measurements. Any readings with deviations of ≥5% were discarded. The particle sizes were converted into volume percent relative to the sum of the detected solids volume material, assuming all of the particles were spherical. This accounted for differences in cell density between the samples included in the comparison, thereby providing a more reliable representation of cell robustness. Cumulative percentage volume was also calculated in order to compare shear effects.

#### 2.8.3 Impurity removal and product yield

DNA and HCP impurities were quantified post the impurity concentration stage as well as in feed samples and post-primary recovery stages. The DNA concentration was determined using a Quant-iT™ PicoGreen® dsDNA Reagent Kit (Invitrogen, Paisley, UK). Due to the high impurity concentrations present in the cell culture test material (CCTM) it was necessary to dilute up to 500 times in order to achieve acceptable readings. In this case the BCA assay (Fisher Scientific, Loughborough, UK) was found to be the most consistent for quantifying relative HCP removal achieved by the various technologies examined. The HCP concentrations were calculated using the following equation:


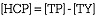
(3)

where TP is the total protein concentration (μg/mL) and TY is the total product concentration in the sample (μg/mL). IgG_1_ concentration was determined using a HiTrap™ Protein G column (GE Healthcare, UK) run on an HPLC system (Agilent Technologies, UK). 100 μL of sample was loaded at <2 g/L at a flowrate of 2 mL/min and eluted at pH 2.8. Detection was carried out at 220 and 280 nm and replicates were typically within 0.1 g/L.

#### 2.8.4 Reducing 2D-PAGE

The concentrated cell culture mimic feed samples pre and post primary recovery were treated using a 2D Clean-Up Kit (GE Healthcare) prior to loading 200 μg of protein resuspended in 125 μg of 7.0 M urea/2.0 M thiourea/4% CHAPS w/v/0.5% carrier ampholyte/0.002% bromophenol blue/40 mM DTT, onto a 7 cm IPGPhor strip holder. The first dimension was then run using a 7 cm IPGPhor strip (pH3-10 Non-Linear, GE Healthcare, UK). Isoelectric focusing was performed using the following settings: controlled temperature at 20°C; ≤50 μA/strip; step 1, step-*n*-hold at 30 V for 12 h; step 2, gradient at 200 V for 45 min; step 3, step-*n*-hold at 500 V for 45 min; step 4, step-*n*-hold at 1000 V for 45 min; step 5, step-*n*-hold at 8000 V for 30 min; step 6, step-*n*-hold at 8000 V until 8000 total volt hours had been reached. The strips were then immediately transferred to be run in the second dimension. The strips were equilibrated in 5 mL of LD sample buffer (Invitrogen, Paisley, UK), containing 1% w/v dithiothereitol, followed by a further equilibration with 5 mL of LDS sample buffer containing 2.5% w/v iodoacetamide. The second dimension was run using 4–20% pre-cast Bis-Tris gels (7.0 × 7.0 × 0.1 cm^3^ ZOOM IPG Well) using the X-Cell SureLock Mini-Gel System (Invitrogen, Paisley, UK). The gels were run at 200 V, 60 W per gel, 45 min running time, using 1 × MES SDS running buffer. The gels were stained using Sypro Ruby™ stain according to the manufacturers protocol. The gels were then scanned using a Typhoon 9400 laser scanner (GE Healthcare, UK) with a 100 μm pixel size and 600 V PMT.

## 3 Results and discussion

**Figure 1:**
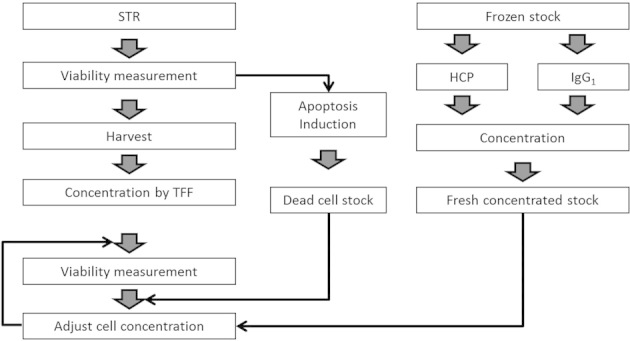
Schematic illustrating the Template methodology used to produce the CCTM, with independent control of cell density, cell viability, HCP, and IgG_1_ concentrations. This methodology was used to achieve a range of high cell densities, viabilities, titer, and HCP concentrations by using cell culture material harvested from an STR at the 5- and 70-L sales at a cell density <10 × 10^6^ cells/mL and a titer of <1 g/L. The cell cultures were harvested and concentrated using TFF, to achieve the target CCTM cell density. When required apoptosis induction was used to generate a dead cell stock which when spiked was able to provide the target viability of the CCTM. HCP and IgG_1_ originating from the GS-CHO cell line were spiked into the CCTM to achieve the target impurity and product concentrations.

A representative set of mimic feeds to primary recovery, termed CCTM were generated to create a wide range of cell culture conditions. The range of conditions generated addresses currently common cell density ranges as well as those reported to be high cell density conditions. The methodology used is summarized in Fig. 1 and involves harvesting low cell density cell culture material produced in a stirred tank bioreactor and concentrating it using a TFF step. An apoptotic cell stock was also created which may be used as a cell stock to achieve required cell viability conditions. Product and HCP stocks were also created by concentrating glutamine synthetase CHO (GS-CHO) cell line derived IgG_1_ and impurities. These were used to achieve target product and impurity concentrations for the selected conditions. The resultant CCTM were characterized in terms of the key variables of cell culture materials when used for primary recovery studies. We analyzed the methodology in terms of significance of the cell culture broth source i.e., the impact of scale on cell sensitivity to shear, as well as the CCTM generation technique and the consistency of the final material.

### 3.1 Impact of the cell material source

**Figure 2:**
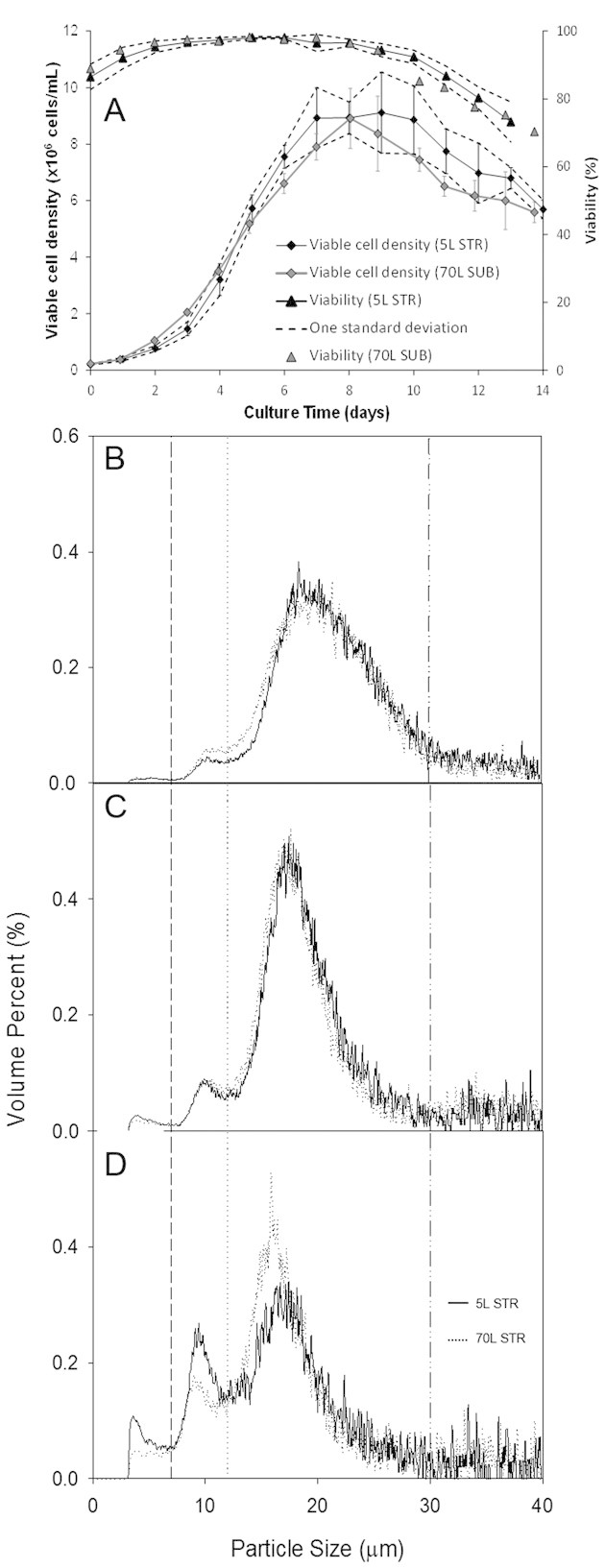
(A) Cell culture growth curves comparing the cell culture growth profiles achieved at the 5-L and the 70-L scales. Average viable cell density and cell viability is shown throughout a 14 day culture period at the two scales. One standard deviation of the repeat 5-L cultures is shown as dotted lines below and above the average values. Dotted lines above and below the average lines indicate ±1 standard deviation of the repeat 5-L cultures and error bars indicate ±1 standard deviation from a single 70-L scale run. (B) Particle size distributions of cell culture material harvested at 70–75% viability at the 5-L and the 70-L scales prior to the application of shear. Average particle size distributions of each sample were obtained (n=5). Measurements where variation was >10% were discarded and the measurement was repeated. (C) Particle size distributions at the two scales post low shear application (0.019 × 106 W/kg). (D) Particle size distributions post high shear application (0.37 × 106 W/kg).

Cell culture material was produced at bench (5-L) and pilot scales (70-L). Cell growth, viability profiles, and the average specific productivity during scale up were predominantly maintained within one standard deviation of the bench scale results (Fig. 2A). Cell shear sensitivity profiles were compared by subjecting cell culture material harvested from both the 5-L STR and the 70-L SUB at equivalent viabilities of ∼75% to conditions of low and high shear (Fig. 2B–D). Prior to the application of shear three populations of cellular material could be observed at both scales. The 0–7 μm region showing small diameter cell debris, 7–12 μm region showing apoptotic cell populations and 12–30 μm region showing live cells. As low shear was applied a shift toward the smaller particle size was observed, as well as an increase in the apoptotic cell population. The decrease in cell size as a response to applied stress conditions has been observed previously [6, 7]. In addition cell damage is expected as shear is applied, causing some of the cells to burst, and subsequently the observed shift in cell size. The change in particle size distribution profiles between no shear and low shear yielded similar profiles at 5- and 70-L scales.

A decrease in live cell population was observed when high levels of shear were applied, along with a concomitant increase in apoptotic and cell debris populations. For the high shear condition, the decrease in the live cell population observed at the 70-L scale was found to be significantly different to that at the 5-L scale (*p* = 0.002). The apoptotic and the debris populations between the scales were found also to be significantly different (*p* < 0.0001). These differences in live, apoptotic and cell debris populations between the scales likely indicates a higher level of susceptibility to shear at the 70-L scale. However, the impact of this difference is not clear and would have to be addressed on a case-by-case basis. Therefore, it is recommended that comparability in shear sensitivity be explored further in those cases where the scale differences are deemed significant.

### 3.2 Generation of mimic feed profiles to primary recovery

**Figure 3:**
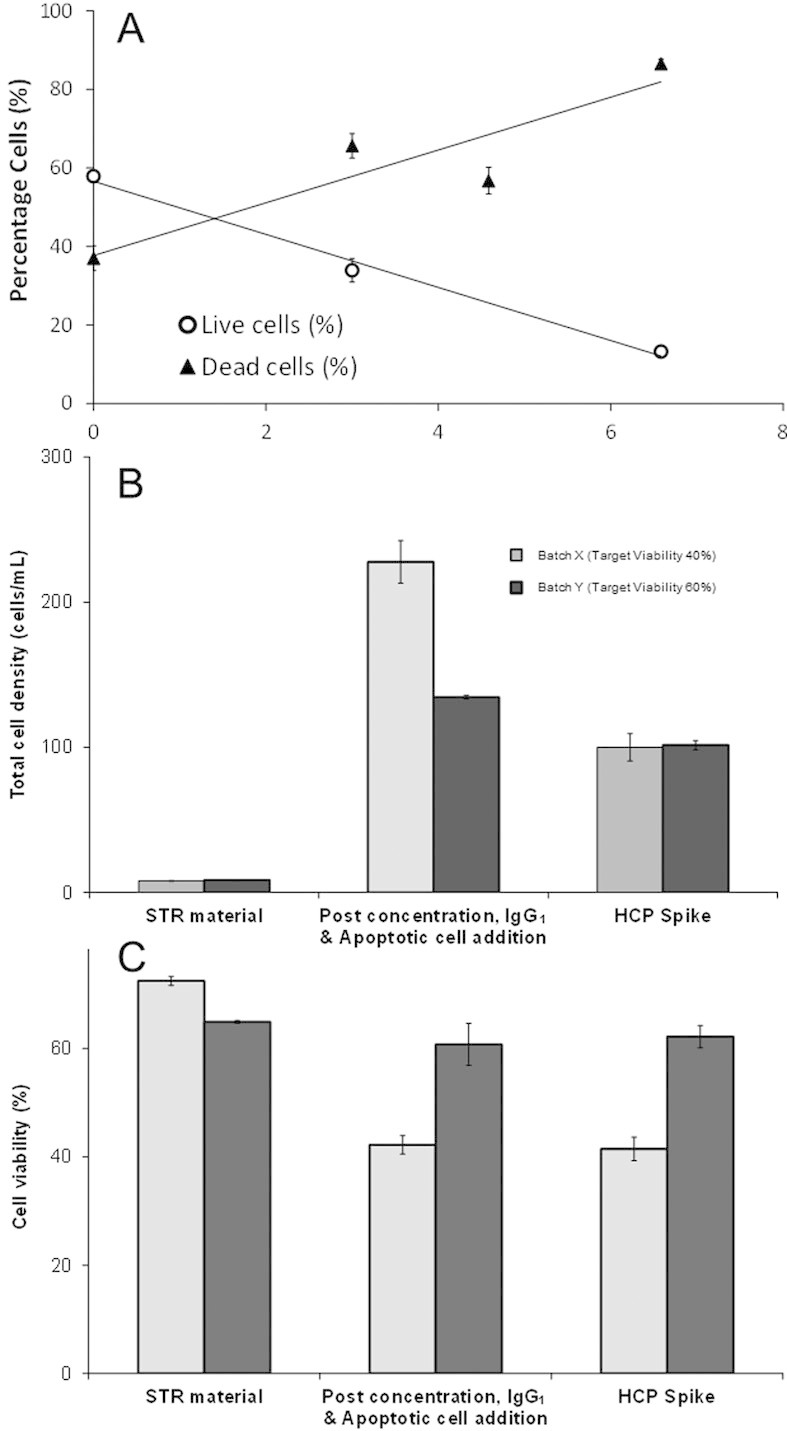
(A) Apoptosis induction during the production of a dead cell stock using cold shock is shown. % live and dead populations were measured using FACS staining using the Annexin V-FITC/7ADD kit. Comparison of total cell density (B) and cell viability (C) achieved in two batches of CCTM generated at the 5-L scale at three key stages of the CCTM methodology. Light grey bars represent Batch X with targeted low viability (40%),and a high cell density of 100 × 10^6^ cells/mL and a concentration factor of ∼28. Dark grey bars represent Batch Y with targeted medium viability (60%), and a high cell density cell density of 100 × 10^6^ cells/mL and a concentration factor of ∼15.

In order to create mimic feed profiles with different cell densities and viabilities, it was necessary to decouple the control of these factors. Different viabilities were achieved through spiking in material from an independently generated apoptotic cell stock. This section illustrates the capabilities of the proposed method for achieving target conditions. To achieve a reduction in the viability of the material generated using the 5-L STRs, apoptosis was induced in a portion on the cell culture, creating an apoptotic cell stock. A similar method has been shown previously to induce apoptosis early in the culture period [17]. A sample of the cell culture material was removed from each culture batch prior to harvest and apoptosis induced in these fractions using cold shock. Apoptosis staining showed a rapid increase in the dead cell population and a decrease in the live cell populations over the course of the following 6 h (Fig. 3A). In addition a migration of the early apoptotic population to the late apoptotic was seen after ∼3.5 h, and subsequent migration of the late apoptotic cell population to the dead cell population at ∼6.4 h post-apoptotic induction (figure not shown). Overall, a decrease to <20% cell viability was achieved within 7 h of apoptotic induction.

Independent control of cell density and viability was demonstrated during the production of the CCTM (Fig. 3B and C). Batches named X and Y cell culture material produced in 5-L STRs were harvested on day 14, at viabilities of 65 and 71% and cell densities of 8 × 10^6^, 8.5 × 10^6^ cells/mL, respectively. Post-processing, cell densities of 100 × 10^6^ cells/mL were achieved for both batches, while simultaneously achieving different target viabilities of 40% for X and 60% for Y. No significant differences in cell viability beyond that attributed to measurement variability was observed during these cell concentration stages.

The generated apoptotic cell stock was spiked into the concentrated material to achieve the target culture viability levels. Due to the difference in volumetric addition of the apoptotic cell stock derived solution a calculated degree of over-concentration was necessary to achieve the final target cell densities for cultures X and Y of 220 × 10^6^ and 130 × 10^6^ cells/mL, respectively. The degree of over-concentration included the volume compensation for the IgG_1_ and the HCP spike volumes. Sample-to-sample variation in both cell density and viability measurements of up to 10% were observed. This was considered to be within the expected measurement variation as dilutions of 50–80 times were required to obtain readings within the detection range of the ViCell™.

### 3.3 Consistency of mimic feed profiles to primary recovery

**Figure 4:**
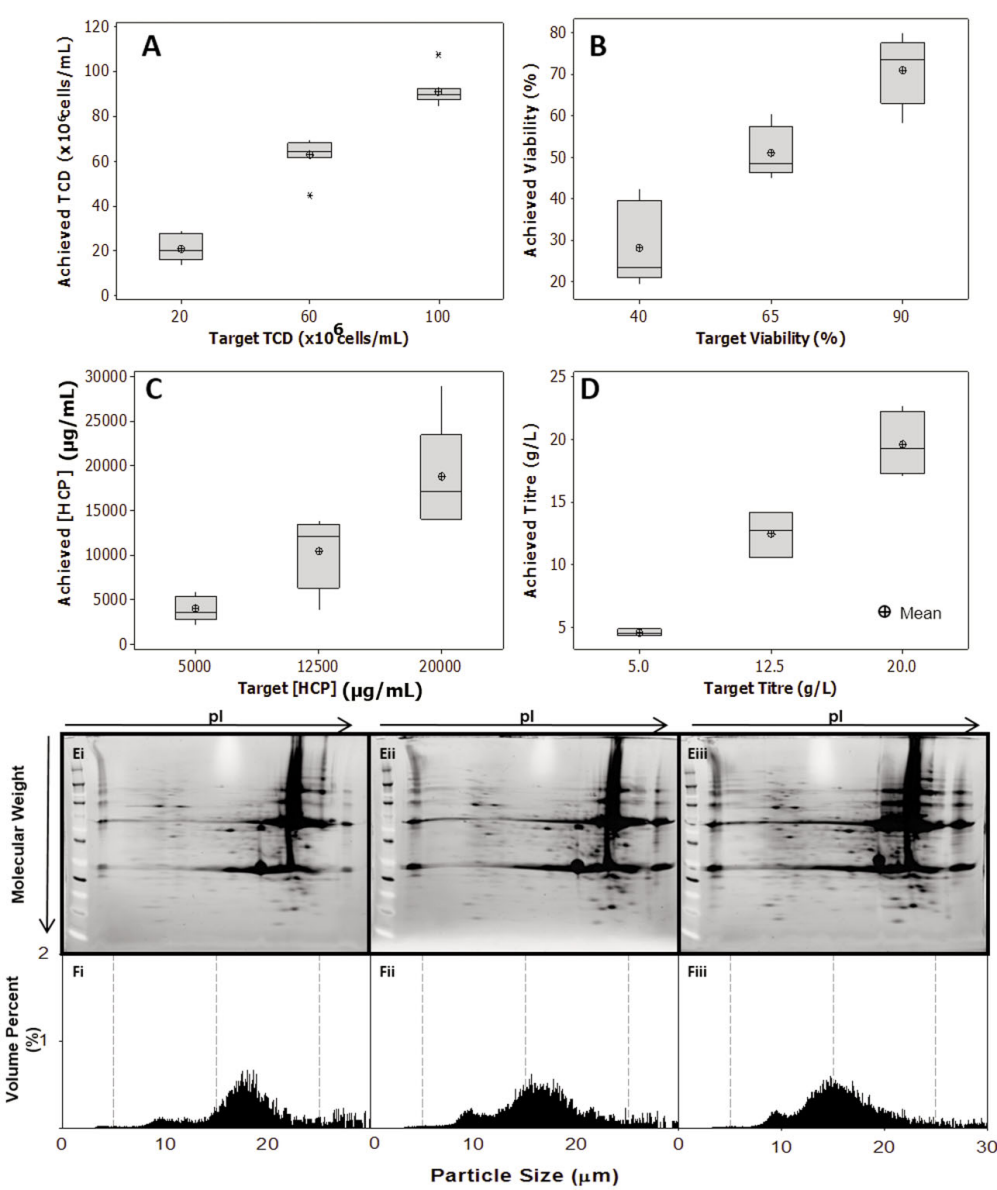
Box plots showing the range in achieved conditions versus the target value for the CCTM. Three target combinations of (A) cell density, (*n* = 8–10), (B) viability, (*n* = 8–10), (C) HCP concentration (*n* = 5–8), and (D) titer (*n* = 5) were tested. These were manufactured using cell culture material generated at 70-L scale. The box represents the 25th hand the 75th percentiles, the median is indicated with a black line, the mean with the star. The whiskers represent data within 1.5 of the interquartile range. (E) Comparsion of CCTM profiles generated using culture material from three 5-L STR cultures in terms of (Ei–Eiii): HCP profiles, including the IgG_1_ product and (Fi–Fiii): equivalent particle size distributions, where % volume fraction of the solids was calculated based on an average measurement of five repeat samples.

The consistency of the CCTM manufacturing methodology was found to vary for each of the target variables. Achieving the target total cell density was found to be relatively reliable, within 10% of the target cell density. Outliers observed at the higher target cell densities of 100 × 10^6^ cells/mL (Fig. 4A) were most likely due to the increase in measurement error when using the ViCell™ for high cell concentrations. Viability showed greater variability at low levels of target cell viability. As cells begin to break down, the accuracy of the trypan blue method decreased, as the apparent total cell count fell. The percentage viability figure consequently became less consistent and representative (Fig. 4B). No dead cell stock addition was required for the 70-L SUB material, as the cell viability reduced during the cell concentration stage of the CCTM production. This reduction in viability during the concentration step is consistent with the particle size distribution data (Fig. 2B–D) suggesting that the material produced at the 5-L scale may be less shear sensitive than that produced at the 70-L scale. In addition, the 70-L scale material was produced during the course of a cell culture starting from day 7 to day 14. Tait et al. [6] has previously shown that day 5–7 cell culture is more susceptible to shear damage and for the susceptibility decreases in the following days. Apoptotic and non-viable cells have been theorized to be more robust due to the increased porosity of the cell membrane. As a result of this reduction limited the viability possible to achieve post-concentration, as the final viabilities were below the target by 15–20%. HCP and titer concentrations were increasingly variable as the target concentrations increased (Fig. 4C and D). This is also likely to be due to the increase in dilution factors required to quantify effectively the HCP and the IgG_1_ concentrations once titer exceeds 10 g/L. The HCP spike consistency was also observed to be consistent. The total HCP concentration was kept within 10% batch-to-batch variation.

IgG_1_ and HCP profiles were also compared in these three CCTM material batches (Fig. 4Ei–Eiii). IgG_1_ and HCPs across the molecular weight and p*I* spectrum were shown to be present in the HCP stock. Some level of inconsistency in the individual isoform volumes were observed, but the overall diversity in the HCP population between the three batches was consistent. By adoption of these methods it was possible to expose the subsequent processing technologies to a consistent IgG_1_ and HCP population within the feed material.

It has been shown previously that toward the end of the culture period mammalian cell culture material typically contains three distinct populations within the particle size distribution: live cell, apoptotic cell, and debris populations [6]. Consistency in particle size distributions of the final CCTM material as well as the HCP population was compared to ensure that the material is to an extent representative of true cell culture material (Fig.4Fi–Fiii 4Fi–Fiii). In terms of particle size distribution, three populations were observed across the three CCTM batches tested. The large peak at >10 μm includes the majority of live cells, the smaller population between 5 and 10 μm in particle size represents the apoptotic cell population and the low volume fraction population with a particle size of <5 μm represents cell debris. However, particles <3 μm were below the detection limit of the instrument, therefore have not been quantified. All three populations are present in the particle size distribution, which is also present in a typical cell culture material originating in an STR. Therefore, the CCTM methodology provided a consistent and representative feed material input to primary recovery operations in terms of particle size distribution.

## 4 Concluding remarks

This paper sets out to develop and demonstrate an experimental methodology for the creation of a mammalian CCTM. Cell density, cell viability, product, and impurity concentrations were manipulated to achieve a range of cell culture test conditions to provide a controlled set of key variables in the cell culture feed to primary recovery. Generation of CCTM material at selected worst case as well as a range of conditions was shown successfully. Key cell culture parameters including cell density, cell viability, product, and HCP concentrations were successfully decoupled. An apoptosis induction by cold shock method was applied to create an apoptotic cell population, which was used to reduce the viability to the selected target conditions. The method was applied to cell culture material originating from bench and pilot scales. Data showed that the accuracy of the target conditions achieved using this method decreased as increasing target concentrations were set. It is likely that this feature is, to an extent, an artifact of some of the analytical techniques applied when assaying very high concentrations. The CCTM methodology, created in this work, was able to preserve the expected three distinct particle size populations for live, apoptotic and debris cells with a low batch-to-batch variation. In addition, the selected HCP stock was found to provide a wide variety of HCPs varying in molecular weight and p*I*, thus providing a basis for determining specific HCP removal capability of a particular unit operation during CCTM application.

Overall, the CCTM method was found to decouple the selected variables in the cell culture feed from downstream operations, while providing consistent material that is representative of typical cell culture material in terms its key features such as particle size distribution and HCP population. These features make this methodology suitable for application in primary recovery and purification unit operation studies.

## Abbreviations

CCTM: cell culture test material
HCP: host cell protein
STR: stirred tank reactor
TFF: tangential flow filtration
